# Ubiquitination mediates Kv1.3 endocytosis as a mechanism for protein kinase C-dependent modulation

**DOI:** 10.1038/srep42395

**Published:** 2017-02-10

**Authors:** Ramón Martínez-Mármol, Katarzyna Styrczewska, Mireia Pérez-Verdaguer, Albert Vallejo-Gracia, Núria Comes, Alexander Sorkin, Antonio Felipe

**Affiliations:** 1 Molecular Physiology laboratory, Departament de Bioquímica i Biomedicna Molecular, Institut de Biomedicina (IBUB), Universitat de Barcelona, 08028 Barcelona, Spain; 2 Clem Jones Centre for Ageing Dementia Research, Queensland Brain Institute, The University of Queensland, Brisbane, Queensland 4072, Australia; 3 Laboratory of Neurophysiology, Universitat de Barcelona and Institut d’Investigacions Biomèdiques August Pi i Sunyer (IDIBAPS), 08036 Barcelona, Spain; 4 Department of Cell Biology, University of Pittsburgh School of Medicine, Pittsburgh, PA, 15261, USA

## Abstract

The voltage-dependent potassium channel Kv1.3 plays essential physiological functions in the immune system. Kv1.3, regulating the membrane potential, facilitates downstream Ca^2+^ -dependent pathways and becomes concentrated in specific membrane microdomains that serve as signaling platforms. Increased and/or delocalized expression of the channel is observed at the onset of several autoimmune diseases. In this work, we show that adenosine (ADO), which is a potent endogenous modulator, stimulates PKC, thereby causing immunosuppression. PKC activation triggers down-regulation of Kv1.3 by inducing a clathrin-mediated endocytic event that targets the channel to lysosomal-degradative compartments. Therefore, the abundance of Kv1.3 at the cell surface decreases, which is clearly compatible with an effective anti-inflammatory response. This mechanism requires ubiquitination of Kv1.3, catalyzed by the E3 ubiquitin-ligase Nedd4-2. Postsynaptic density protein 95 (PSD-95), a member of the MAGUK family, recruits Kv1.3 into lipid-raft microdomains and protects the channel against ubiquitination and endocytosis. Therefore, the Kv1.3/PSD-95 association fine-tunes the anti-inflammatory response in leukocytes. Because Kv1.3 is a promising multi-therapeutic target against human pathologies, our results have physiological relevance. In addition, this work elucidates the ADO-dependent PKC-mediated molecular mechanism that triggers immunomodulation by targeting Kv1.3 in leukocytes.

Voltage-dependent potassium channels participate in propagating electrical impulses in excitable cells such as myocytes and neurons^1^. In addition, ion channels control leukocyte physiology^2^. The voltage-dependent potassium channel Kv1.3 modulates membrane potential and drives Ca^2+^ influx in immune cells, including T-cells, dendritic cells and macrophages, thereby regulating activation, proliferation and migration^3^. Altered Kv1.3 expression is associated with multiple autoimmune diseases and changes in sensory discrimination. Therefore, Kv1.3 is an emerging therapeutic target in T-cell-mediated diseases such as multiple sclerosis, rheumatoid arthritis, type 1 diabetes mellitus and psoriasis^3^.

Kv1.3 signaling relies on the activity, abundance and proper localization of channels at the cell surface. In this respect, Epidermal growth factor receptor (EGFR) activity regulates Kv1.3 by both tyrosine phosphorylation and ERK1/2-dependent endocytosis, with consequences for neuronal fate^4^,^5^. Kv1.3 is also regulated by PKC, modulating T-cell activation^6^. In this context, adenosine (ADO), a potent endogenous anti-inflammatory mediator in leukocytes, activates PKC-dependent signaling pathways^7^,^8^. Also physiologically relevant is the spatial regulation of ion channels within specific membrane lipid raft domains^9^. Raft microdomains are cell platforms that concentrate signaling molecules, such as PKC and their targets^9^,^10^. Lipid rafts recruit Kv1.3 in macrophages and in the immunological synapse (IS) of cytotoxic T lymphocytes^11^. The localization of Kv1.3 in rafts and caveolae is dependent on the accessibility of a caveolin-binding domain near the T1 domain and of the Kvβ subunit recognition motif at the N-terminal of the channel^12^. Evidence demonstrates that the control of Kv1.3 surface abundance takes place at multiple stages, balancing forward trafficking mechanisms to the cell membrane and the internalization of fully functional channels^4^,^13^. These results strongly support the idea that the prevalence of Kv1.3 channels at the membrane surface has enormous consequences for cell physiology.

In the present study, we show the clathrin-mediated PKC-induced internalization of Kv1.3. ADO, activating PKC, down-regulates Kv1.3 by increasing the endocytosis and lysosomal degradation of the channel. This mechanism is mainly mediated via the ubiquitination of Kv1.3 by the E3 ubiquitin ligase Nedd4–2 (neural precursor cell expressed, developmentally downregulated 4–2) and is essential for fine-tuning the immunological response. Moreover, PSD-95 protects Kv1.3 from the PKC-induced internalization and ubiquitination by inducing the clustering of the channel in membrane raft microdomains. This PKC-dependent Kv1.3 downregulation is crucial for understanding the anti-inflammatory effect of ADO in leukocytes. Overall, our results elucidate the complex interactions between Kv1.3 and scaffolding proteins within the channelosome, which are essential for the proper establishment of the immunological synapse between T lymphocytes and antigen-presenting cells during the adaptive immune response.

## Results

### Adenosine hampers the LPS-dependent activation of macrophages and dendritic cells concomitantly with a down-regulation of Kv1.3

**Figure 1:**
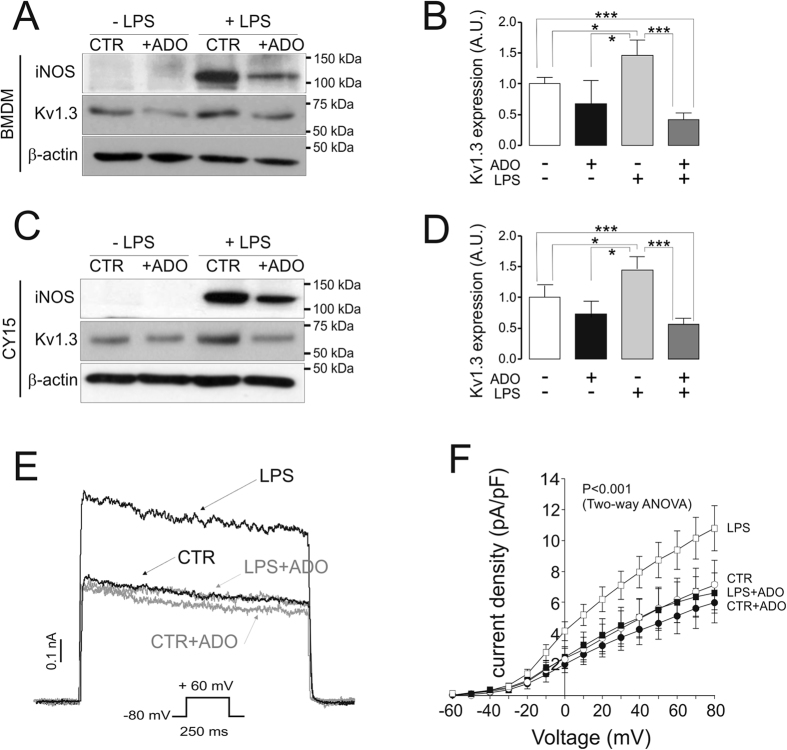
Adenosine counteracts the LPS-dependent activation and Kv1.3 induction in macrophages and dendritic cells. Murine bone marrow-derived macrophages (BMDM) and CY15 dendritic cells were incubated with (+) or without (−) LPS (100 ng/ml) in the presence (+ADO) or the absence (control, CTR) of 200 μM adenosine for 24 h. (A-D) Cells were lysed, and protein expression of Kv1.3 and iNOS was analyzed. (**A**,**B**) BMDM; (**C**,**D**) CY15 dendritic cells. (**A**,**C**) Representative western blots; (**B**,**D**) Kv1.3 expression in arbitrary units (A.U.). β-Actin was used as a control reference. Values are mean ± SE of 4–5 independent experiments. Statistical analysis by One-Way ANOVA (P < 0.001) with a Tuckey post-test (*, p < 0.05; ***, p < 0.001). (**E**) CY15 cells were held at −80 mV, and voltage-dependent K^+^ currents were elicited by a 250 ms depolarizing pulse from −80 mV to +60 mV. Black traces, CY15 cells in the absence of ADO; grey traces, cells in the presence of ADO. (**F**) Current density vs. voltage for outward K^+^ currents in CY15 cells. Currents were elicited by 250 ms pulses from −60 mV to +80 mV in 10 mV steps. Circles, control cells in the absence (○) or the presence (●) of ADO. Squares, LPS-treated cells in the absence (□) or the presence (■) of ADO. Statistical analysis was performed by Two-Way ANOVA (p < 0.001, LPS vs control, CTR + ADO and LPS + ADO) with a Bonferroni post-test (p < 0.05, LPS vs all other groups at −10 mV; p < 0.01, LPS vs all other groups at 0 mV; p < 0.001, LPS vs all other groups from 10 to 80 mV). Values are shown as the mean ± SE (n = 5–10 independent cells).

Kv1.3 is crucial during proliferation and activation in leukocytes. Bacterial lipopolysaccharide (LPS) activates macrophages, thereby inducing the expression of iNOS (inducible nitric oxide synthase). LPS also increases Kv1.3 activity through transcriptional and translational controls. Pharmacological blockage of Kv1.3 decreases the iNOS expression, demonstrating that this channel participates in the LPS-dependent macrophage activation^14^. ADO, an endogenous anti-inflammatory agent, modulates various functional activities such as the antimicrobial responses of immune cells^15^. In this context, we cultured murine bone marrow derived macrophages (BMDM) and CY15 cells, a histiocytic tumor cell line that phenotypically mimics immature dendritic cells, with LPS in the presence of ADO. LPS triggered iNOS expression in both mononuclear phagocyte cell models (Fig. 1A,C). However, ADO hampered iNOS induction as well as the LPS-dependent Kv1.3 increase (Fig. 1A–D). ADO also slightly, but statistically non-significant, decreased the basal levels of Kv1.3 in control cells (BMDM, 2 over 4 experiments; CY15, 3 over 5 experiments). These effects were also observed in the Kv currents elicited from CY15 dendritic cells (Fig. 1E,F). Thus, while LPS increased outward K^+^ currents, the presence of ADO halted this induction (Fig. 1E,F). Kv currents were slightly, but statistically non-significant, diminished by ADO in control cells, which is consistent with the minor effects on Kv1.3 (Fig. 1D) and a notable contribution of Kv1.5 in dendritic cells^16^,^17^, which expression was not altered under any situation.

### Adenosine triggers PKC-dependent Kv1.3 endocytosis in HEK-293 cells

ADO, which stimulates PKC and PKA^18^, decreased the expression and activity of Kv1.3 in activated macrophages and CY15 dendritic cells. Although PKC mostly triggers internalization of channels and transporters^19^,^20^,^21^,^22^, ADO stabilizes KATP channels at the membrane surface in a PKC-dependent manner^23^. With this debate in mind, we investigated whether the modulation of Kv1.3 was consequence of a specific PKC-mediated endocytosis. HEK-293 cells, similarly to macrophages^24^,^25^,^26^, endogenously express the A_2B_ subtype of adenosine receptors^27^. Therefore, this cell line is a good model for dissecting the ADO-dependent PKC signaling.

**Figure 2:**
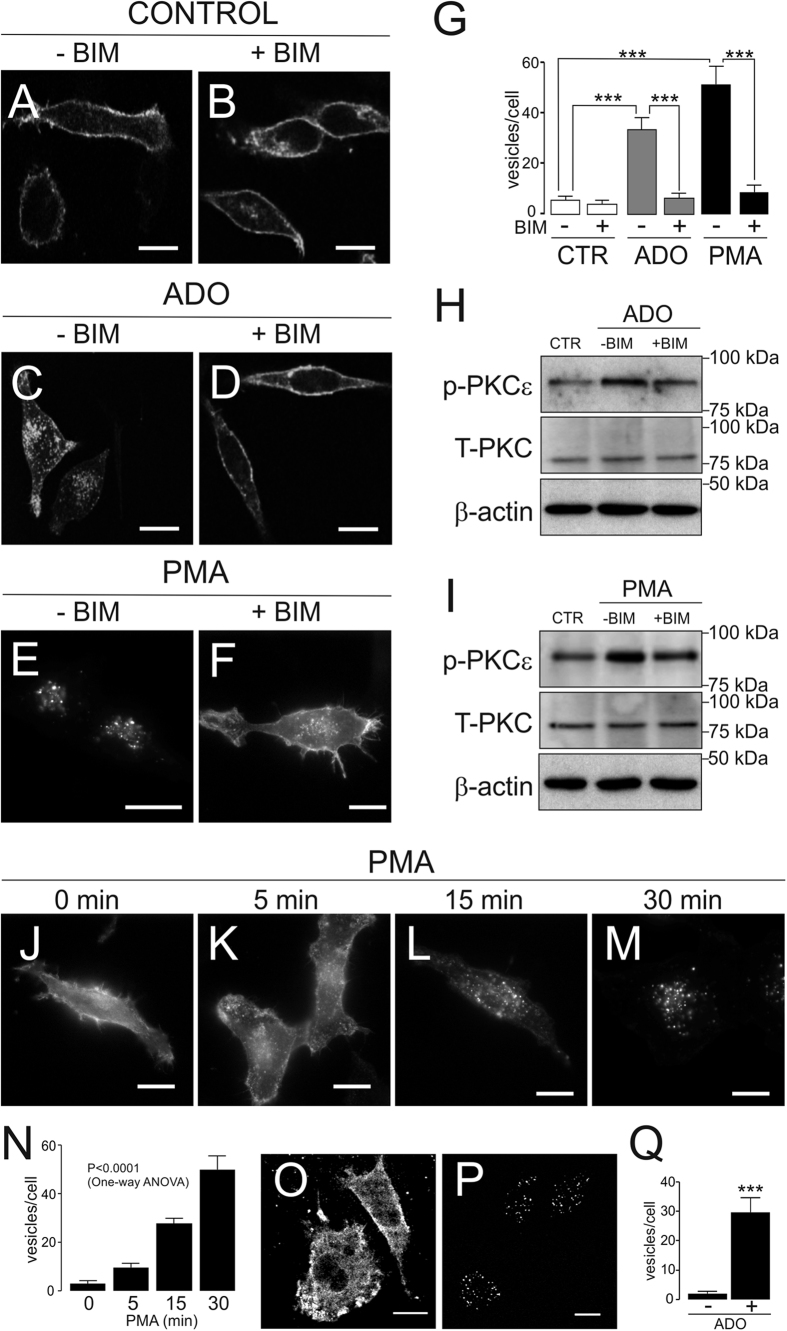
ADO- and PMA-dependent PKC stimulation triggered Kv1.3 endocytosis. Representative confocal images of HEK-293, with stable expression of Kv1.3-YFP, and CY15 dendritic cells incubated in the presence or the absence of 200 μM Adenosine and 1 μM PMA at the indicated times. (**A**–**F**) HEK-293 cells cultured for 30 min at 37 °C in the absence (**A**,**B**) or the presence of ADO (**C**,**D**) and PMA (**E**,**F**) with (**B**,**D**,**F**) or without (**A**,**C**,**E**) 1 μM BIM. (**G**) Quantification of Kv1.3 intracellular vesicles from representative images on panels A–F. Values are mean ± SE of n = 20–25 cells. A One-Way ANOVA analysis revealed differences vs treatment (p < 0.001). A further Tuckey post-test among groups is indicated (***, p < 0.001). (**H**,**I**) Adenosine and PMA-dependent PKCε phosphorylation (p-PKCε. HEK cells were incubated during 30 min in the absence (CTR, Control) or in the presence (ADO and PMA) of any insult without (−) or with (+) BIM. β-Actin was used as a reference control. Note that total PKC abundance (T-PKC) was not altered. (**J**–**M**) Time-dependent Kv1.3 endocytosis in the presence of PMA. (**N**) Quantification of Kv1.3 intracellular vesicles from representative images on panels J–M. Values are mean ± SE of n = 20–25 cells. Statistical analysis was performed by One-Way ANOVA (***, p < 0.0001 vs time). (**O**,**P**) Representative images of Kv1.3 staining in CY-15 dendritic cells incubated during 30 min without (**O**) or with (**P**) 200 μM ADO. (**Q**) Quantification of intracellular vesicles from representative images on panels O and P. Values are mean ± SE of n = 10–15 cells. ***, p < 0.001 vs the absence of ADO (–) by the Student’s *t* test. Bars represent 10 μm.

**Figure 3:**
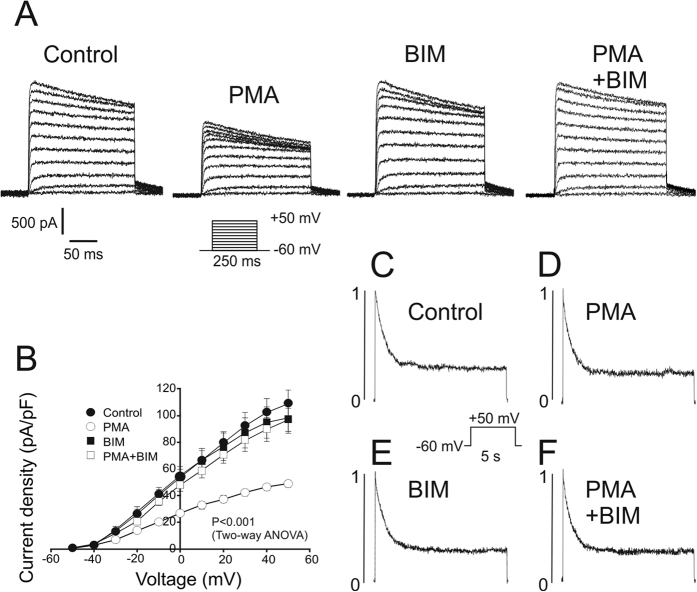
PKC-dependent stimulation decreases Kv1.3 currents. HEK-293 cells stably transfected with Kv1.3-YFP were incubated for 30 min with or without 1 μM PMA at 37 °C in the presence or the absence of 1 μM BIM. Cells were held at −60 mV, and voltage-dependent K^+^ currents were elicited by 250 ms depolarizing pulses from −60 mV to +50 mV in 10 mV steps. (**A**) Representative current traces from cells preincubated with or without BIM in the presence or the absence of PMA. (**B**) Current density versus voltage plot of K^+^ currents. ●, control cells with no treatment; ○, PMA-treated cells; ■, cells preincubated with BIM; □, cells treated with PMA and preincubated with BIM. Values are shown as the mean ± SE (n = 6–10 independent cells). Statistical analysis was performed by Two-Way ANOVA (p < 0.001 PMA vs Control, BIM and PMA + BIM) with a Bonferroni post-test (p < 0.01, PMA vs all other groups at −20 mV; p < 0.001, PMA vs all other groups from −10 to 50 mV). (**C**–**F**) C-type inactivation of Kv1.3 currents. Cells were held at –60 mV, and a 5 s depolarizing pulse of +50 mV was applied. Representative traces from 6–8 independent cells are shown and normalized to 1 in order to better compare the inactivation kinetics. (**C**) Control, 720 ± 14 ms. (**D**) PMA, 698 ± 14 ms. (**E**) BIM, 743 ± 30 ms. (**F**) PMA ± BIM, 741 ± 11 ms. No statistical significance was found by Mann-Withney U test.

We further analyzed the participation of a PKC-dependent mechanism by using the PKC agonist phorbol 12-myristate 13-acetate (PMA). The presence of the PKC inhibitor bisindolylmaleimide (BIM) hampered the internalization of channels (Fig. 2A–D,G), indicating that ADO triggered Kv1.3 endocytosis via stimulation of PKC. Similarly, specific PMA-dependent PKC activation also promoted the redistribution of Kv1.3 from the cell surface to vesicular structures (Fig. 2E–G). BIM abolished this effect, indicating that, similar to ADO-induced endocytosis, PMA-associated endocytosis was dependent on PKC (Fig. 2B,D,F,G). This mechanism was indeed concomitant to an activation of the PKC. We analyzed the phosphorylation of PKCε because this isoform participates in TNF-α-dependent pro-inflammatory responses in HEK cells^28^ and during the LPS-induced and MCSF-dependent activation in macrophages^29^,^30^,^31^. ADO (Fig. 2H) and PMA (Fig. 2I) augmented the phosphorylation of PKCε (~2 fold increase), without changes in total PKC. Again BIM effectively hampered (~50% decrease) PKC phosphorylation in both cases. Next, we monitored the time-course of Kv1.3 distribution under persistent PKC activation. PMA steadily induced the endocytosis of Kv1.3 (p < 0.0001, One-way ANOVA), which was almost completely internalized within 30 minutes (Fig. 2J–N). Supporting HEK 293 cells, similar mechanisms functioned in mononuclear phagocytes (see Fig. 1), because a 30 min incubation with ADO (Fig. 3O–Q) vesicularized Kv1.3 in CY15 dendritic cells.

The “antibody-feeding assay” was essential to unequivocally decipher the PKC-dependent endocytosis of the dopamine transporter (DAT) and the EGFR-dependent internalization of Kv1.3^4^,^21^. HA-Kv1.3-YFP channels that simultaneously contain an extracellular HA tag and an intracellular YFP fluorophore were expressed in HEK-293 cells. Total (YFP) and extracellular (Cy5) Kv1.3 channels were observed under non-permeabilizing conditions. In the absence of PMA, cells showed surface HA-Kv1.3-YFP channels when stained with Cy5-labeled anti-HA antibodies (Fig. S1A,B). In addition, some Cy3-labeled intracellular vesicles were also observed (Fig. S1C). Because Kv1.3 recycling is rapid, this would correspond to constitutively internalized HA-Kv1.3-YFP channels in early endosomes. PMA increased intracellular Kv1.3-containing Cy3-stained vesicles (Fig. S1G). Intracellular channels were inaccessible to Cy5-labeled secondary antibodies applied to non-permeabilized cells, both a decrease in extracellular Cy5 channels and an increase in internal Cy3-stained channels were observed upon PKC activation (Fig. S1E–H).

We next analyzed whether the Kv1.3 internalization was associated with changes in Kv currents in Kv1.3-YFP stable HEK-293 cells. Depolarizing pulses elicited K^+^ currents, and 30 min incubation with PMA reduced those currents by 49%. BIM counteracted this effect, demonstrating a specific dependence on the PKC stimulation (Fig. 3A). The I–V plots showed that PKC activation efficiently reduced current densities at all activation voltages (Fig. 3B) (p < 0.001, two-way ANOVA). We further analyzed the specific Kv1.3 C-type inactivation (Fig. 3C–F). This hallmark, which is the result of the cooperative interaction of all subunits within the complex, remained unaltered^32^. Thus, the τ of current decay (in ms) was similar being 720 ± 14, 698 ± 14, 742 ± 30 and 741 ± 11 for Control (Fig. 3C), PMA (Fig. 3D), Control + BIM (Fig. 3E) and PMA + BIM (Fig. 3F) respectively. Taken together, these results further supported the down-regulation of Kv1.3, mostly governed by a PKC-mediated decrease of channel subunits at the cell surface, rather than major changes in Kv1.3 biophysics upon PKC activation.

### PKC-dependent Kv1.3 endocytosis is a clathrin-mediated mechanism

**Figure 4:**
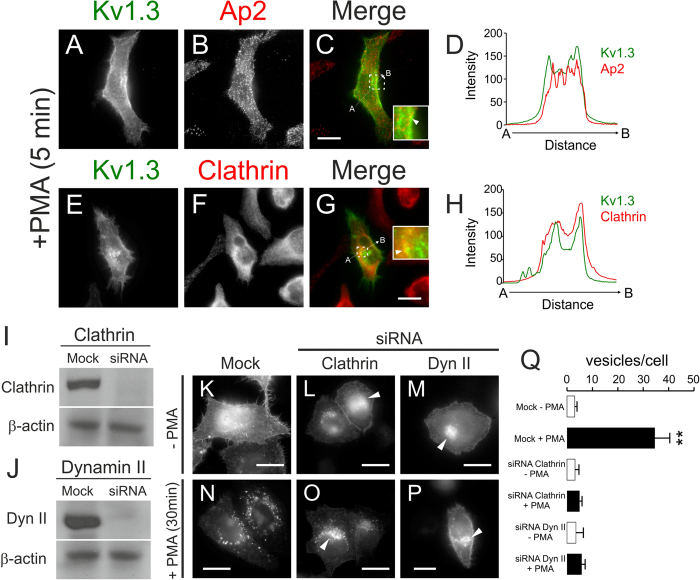
PMA-dependent Kv1.3 internalization is governed by clathrin-mediated endocytosis. Confocal images and western blot analysis of cells stably transfected with Kv1.3-YFP. (**A**–**H**) Cells were stained for AP2 (**A**–**D**) and Clathrin (**E**–**H**) after 5 min of 1 μM PMA incubation at 37 °C. Insets in merged panels highlight the intracellular colocalization in discrete vesicles pointed by arrowheads. Arrowheads in C, G indicate channels that colocalized with AP2 and Clathrin, respectively. (**D**,**H**) Histogram showing the results of the pixel-by-pixel analysis of the section indicated by the arrow in the merged images (n > 25 independent cells for each condition). Color code in panels: Kv1.3 (green), AP2/Clathrin (red), merge (yellow). (I and J) Cells were transfected with siRNAs scramble (Mock) or with siRNAs targeting Clathrin Heavy Chain (Clathrin) and Dynamin II (Dyn II). Cell lysates were blotted with antibodies against Clathrin, Dyn II, and β-actin. Western blots demonstrated the depletion of Clathrin (**I**) and Dyn II (**J**). (**K**–**Q**) Cells mock transfected (**K**,**N**) or transfected with siRNAs targeting Clathrin (**L**,**O**) or Dyn II (**M**,**P**) were incubated with (+PMA) or without (−PMA) 1 μM PMA for 30 min. Arrowheads in L, M, O, P highlight intracellular accumulated Kv1.3. Bars represent 10 μm. (**Q**) Quantification of intracellular vesicles from cells in panels K-P in the absence (white bars) or in the presence (black bars) of PMA. Values are the mean ± SE of n = 24–30 cells. **, p < 0.01 vs Mock −PMA (Student’s t test).

We next dissected the mechanisms of PKC-dependent Kv1.3 endocytosis (Fig. 4). After 5 minutes of PMA incubation, Kv1.3 colocalized with AP2 (Adaptor-related Protein complex 2) and Clathrin, components of the clathrin-coated pit (CCP) internalization machinery (Fig. 4A–H). Thus, a pixel by pixel analysis indicated that Kv1.3 followed a intracellular pattern that overlaid with that of Clathrin and AP2 (Fig. 4D,H). To further decipher this mechanism, stable Kv1.3 HEK cells were transfected with siRNAs against dynamin II (Dyn II) and Clathrin (Fig. 4I–Q), both crucial for the EGFR-mediated clathrin-dependent endocytosis of Kv1.3^4^. Under effective Clathrin and Dyn II depletion (Fig. 4I,J), cells were incubated without (Fig. 4K–M) or with (Fig. 4N–P) PMA for 30 min. Unlike mock treated cells, no significant PMA-dependent internalization of Kv1.3 was observed in Clathrin- or Dyn II-depleted cells (Fig. 4Q). Clathrin and Dyn II also participate in the exocytic pathway of membrane proteins^33^; therefore, some perinuclear Kv1.3 accumulation was observed (arrowheads in Fig. 4L,M,O,P). Altogether, this evidence supports that CCP-mediated endocytosis was the main pathway for PKC-dependent Kv1.3 internalization.

Kv1.3 is targeted to sphingolipid- and cholesterol-enriched lipid rafts^34^. Caveolae/raft-dependent endocytosis upon PKC stimulation may be relevant for some ion channels located in such specialized microdomains^35^. Therefore, we investigated whether this endocytic mechanism was also involved in the PKC-dependent Kv1.3 internalization. Filipin and Nystatin inhibit lipid raft- and caveolae-dependent internalization^36^. However, neither treatment affected the PKC-dependent Kv1.3 endocytosis (Fig. S2A–C,E–G). In addition, lipid rafts are profoundly affected by cholesterol-modifying agents such as methyl-β-cyclodextrin (MβCD)^34^. Although MβCD alters the raft targeting of Kv1.3, it did not affect the endocytosis of Kv1.3 (Fig. S2D,H). To further exclude the involvement of caveolae-dependent internalization, we used a caveolin 1-null (Cav1^−^) HEK-293 cell line^12^. Whether Cav1^−^ cells were treated with or without MβCD, Kv1.3 always underwent endocytosis in the presence of PMA (Fig. S2I–L). Therefore, PKC-dependent Kv1.3 endocytosis is a clathrin-mediated but caveolin-independent mechanism.

### PSD-95 association stabilizes Kv1.3 at membrane raft microdomains and prevents PKC-mediated channel endocytosis

**Figure 5:**
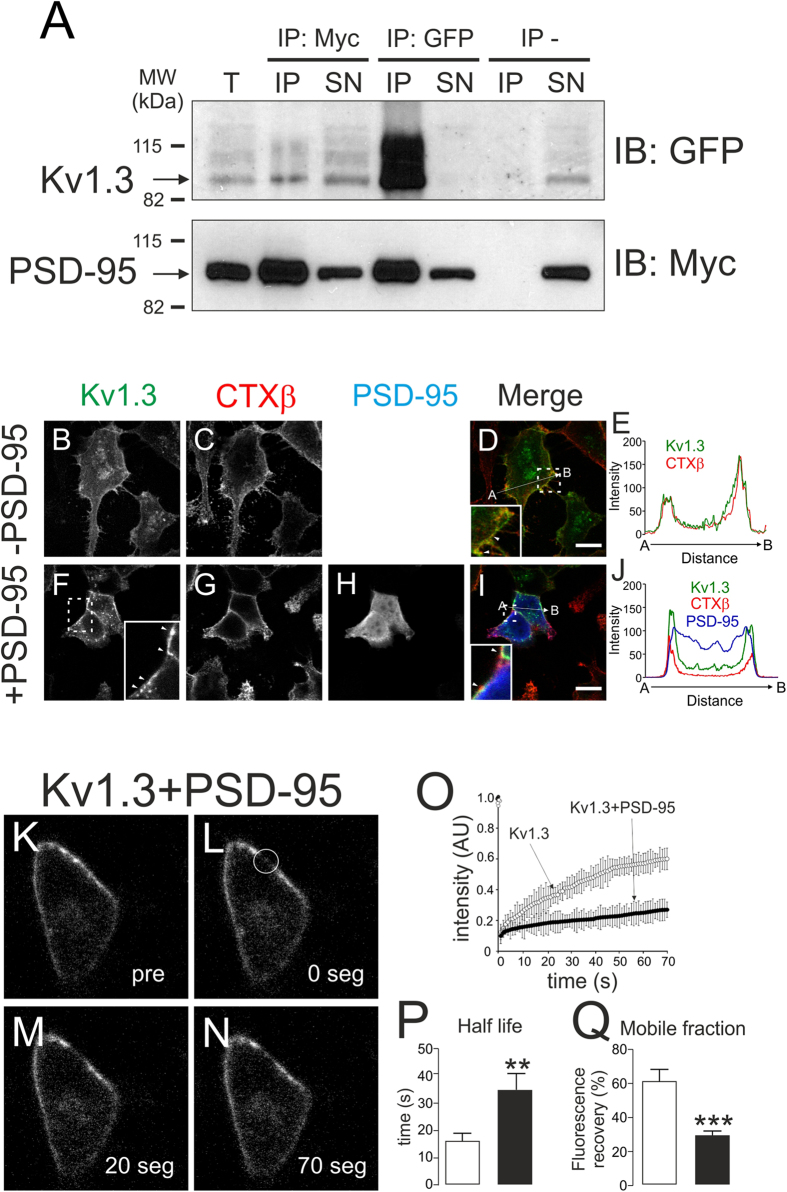
Interaction with PSD-95 recruits Kv1.3 into less-mobile membrane structures. HEK-293 cells with stable expression of Kv1.3-YFP were cotransfected with PSD-95-myc for 24 h. (**A**) Co-immunoprecipitation of Kv1.3 and PSD-95. Cell lysates were immunoprecipitated (IP) for PSD-95 (Myc) and Kv1.3 (GFP), and membranes were blotted (IB) for Kv1.3 (GFP, top panel) and PSD-95 (Myc, bottom panel). T, total lysates; IP, immunoprecipitates; SN, supernatants; IP−, absence of antibody. (B-J) PSD-95 recruits Kv1.3 into discrete membrane clusters. (**B**–**E**) Kv1.3 colocalized evenly with cholera toxin β subunit (CTXβ), which stains lipid raft microdomains. (F-J) PSD-95 recruited Kv1.3 to discrete cell surface clusters that are also positive for CTXβ. Insets in D, F and I highlight areas of interest. Arrowheads indicate clusters (**F**) and colocalization spots (**I**). Bars represent 10 μm. (**E**,**J**) Histogram showing a pixel-by-pixel analysis of the section indicated by the arrow in the merged images. Color code in panels: green, Kv1.3; red, CTXβ; blue, PSD-95; yellow, merge of Kv1.3 and CTXβ; white, triple merge of Kv1.3, CTXβ and PSD-95. (**K**–**Q**) FRAP experiments indicate that PSD-95 targets Kv1.3 to less-mobile membrane microdomains. (**K**–**N**) Representative FRAP images of Kv1.3 in the presence of PSD-95. The membrane surface was bleached at discrete ROIs (regions of interest) in the circle (**L**), and the fluorescence recovery was analyzed at the indicated times (in seconds). Pre, pre-bleaching. (**O**) Intensity of Kv1.3-YFP recovery after photobleaching, in arbitrary units (AU), versus time (s) in the absence (○) or the presence (●) of PSD-95. (**P**) Half time (s) and (**Q**) Mobile fraction (%) analysis of the fluorescence recovery after photobleaching. Values are the mean ± SE of n >25 cells. White bars, Kv1.3; black bars, Kv1.3 + PSD-95. **, p < 0.01; ***, p < 0.001 vs Kv1.3 in the absence of PSD-95 (Student’s t test).

PKC and Kv1.3 are recruited into the IS, fine-tuning the T-cell activation^10^,^11^. Moreover, the IS concentrates lipid raft microdomains as signaling platforms^10^. Kv1.3 interacts with proteins from the MAGUK family such as PSD-95, which recruits the channel into the IS^37^. Impaired IS localization of Kv1.3 is associated with lupus erythematosus^38^. Therefore, PKC-mediated internalization of Kv1.3 would affect the immune response. Thus, we analyzed whether PSD-95 protects Kv1.3 against PKC-dependent endocytosis. PSD-95 associated with Kv1.3 in HEK-293 cells (Fig. 5A), affecting the channel membrane distribution (Fig. 5B–J). Kv1.3 evenly decorated the cell membrane (Fig. 5B,F), whereas the presence of PSD-95 induced the formation of discrete clusters that colocalized with lipid raft markers (Fig. 5F–J). To further demonstrate the effect of PSD-95 on the cell membrane targeting of Kv1.3, we performed fluorescence recovery after photobleaching (FRAP) experiments in the presence of PSD-95 (Fig. 5K–Q). FRAP values indicated that the presence of PSD-95 increased about 2-fold the half-life (p < 0.01) and decreased by 50% the mobile fraction (p < 0.001) of Kv1.3 (Fig. 5O–Q). These results suggested that PSD-95 recruited Kv1.3 to less-mobile membrane microdomains.

**Figure 6:**
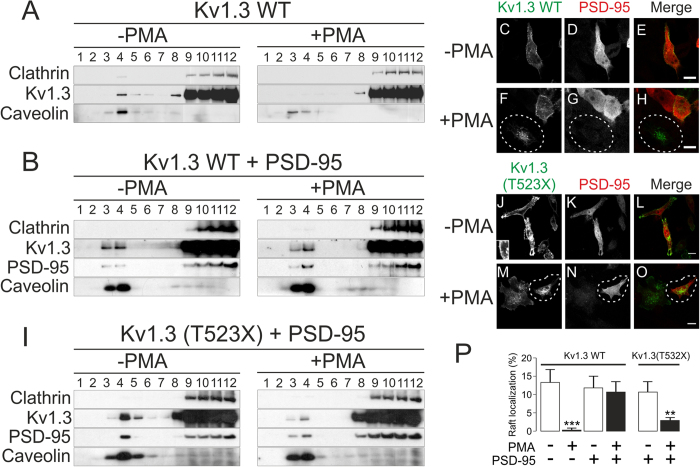
PSD-95 stabilizes Kv1.3 in lipid rafts and protects the channel against PMA-induced endocytosis. HEK-293 cells stably expressing Kv1.3-YFP wild-type (WT) or transiently transfected with Kv1.3(T523X)-YFP were cultured without (left panels) or with (right panels) 1 μM PMA for 30 min at 37 °C, in the absence (**A**) or presence (**B**,**I**) of PSD-95-myc. After the treatment, lipid raft localization and PMA-induced internalization of Kv1.3 were analyzed. (**A**) Representative lipid raft localization of Kv1.3 WT in the presence (+) or the absence (−) of PMA. A continuous sucrose gradient from low-density (1) to high-density (12) fractions was performed. Caveolin and clathrin indicated lipid raft and non-lipid raft fractions, respectively. (**B**) Lipid raft localization of Kv1.3 WT and PSD-95 in the presence (+) or the absence (−) of PMA. (**C**–**H**) PSD-95 protected Kv1.3 WT against PMA-induced internalization. Cells incubated in the absence (**C**–**E**) or the presence (**F**–**H**) of PMA. Note that Kv1.3 in the circled cell in panels F–H did not express PSD-95 and underwent internalization. Color code in merge panels: green, Kv1.3-YFP; red, PSD-95-myc; yellow, colocalization. (**I**–**O**) The mutant Kv1.3(T523X)-YFP channel was not protected by PSD-95 against lipid raft delocalization or PMA-induced endocytosis. (**I**) Lipid raft localization of Kv1.3(T523X) and PSD-95 in the presence (+) or the absence (−) of PMA. (**J**–**O**) PSD-95 did not protect Kv1.3(T523X) against PMA-induced internalization. Cells incubated in the absence (J-L) or the presence (**M**–**O**) of PMA. Inset in panel J shows that, like Kv1.3 WT (**C**), Kv1.3(T523X) is targeted evenly to the cell surface in the absence of PMA. Note that the presence of PSD-95 in the highlighted cell in panels M-O did not prevent Kv1.3 internalization. Color code in merge panels: green, Kv1.3-YFP; red, PSD-95-myc; yellow, colocalization. Bars represent 10 μm. Confocal images are representative from >25 cells analyzed in 3–5 independent experiments. (**P**) Quantitative analysis of the PSD-95 effects on the lipid raft expression of Kv1.3 WT and Kv1.3(T523X) in the presence or the absence of PMA. Floating raft fractions were defined by the expression of Caveolin. Values are the mean ± SE of 4 independent experiments. White bars, absence of PMA; black bars, presence of PMA. Statistical analysis was performed by using One-Way ANOVA with a Tuckey post-test. **, p < 0.01 Kv1.3(T532X) + PMA vs Kv1.3(T532X) − PMA; ***, p < 0.001 Kv1.3 WT + PMA vs Kv1.3 WT − PMA.

Because PSD-95 redistributed Kv1.3 at the cell surface, we next analyzed whether PSD-95 could modify the PKC-dependent Kv1.3 internalization. PMA shifted Kv1.3 out of low-buoyancy fractions, suggesting that the CCP internalization delocalized the channel from lipid raft microdomains (Fig. 6A,P). However, PSD-95 stabilized Kv1.3 in floating fractions in the presence of PMA (Fig. 6B,P). In addition, PSD-95 protected Kv1.3 against the PKC-dependent internalization triggered by PMA (Fig. 6C–H). While Kv1.3 was endocytosed in PSD-95-negative cells (circled cell, Fig. 6F–H), Kv1.3 remained at the surface in the presence of PSD-95 (unmarked cell, Fig. 6F–H).

To further demonstrate that PSD-95 protected Kv1.3 against PKC-dependent endocytosis, we used the HA-Kv1.3-YFP channel. In this experiment, while PMA elevated Kv1.3-YFP-positive intracellular vesicles, it almost completely abolished Kv1.3-HA extracellular staining (Fig. S3A–F). However, the presence of PSD-95 prevented the loss of the Kv1.3 extracellular HA signaling (Fig. S3G–N). In the absence of PSD-95, PKC activation triggered strong colocalization of Kv1.3 with EEA1 (Early Endosomal Antigen 1) (circled cell, Fig. S3S–U; inset, Fig. S3V). However, PSD-95 reduced the colocalization between Kv1.3 and EEA1, further demonstrating that the MAGUK protein stabilized the channel at the cell surface.

Kv1.3 interacts with PSD-95 through a C-terminal PDZ-binding domain, defined by the last three residues of the channel (Thr^523^-Asp^524^-Val^525^)^37^. Therefore, we used Kv1.3(T523X), a truncated channel that lacks this motif and thus cannot bind PSD-95^37^. Consequently, PSD-95 did not protect Kv1.3(T523X) from targeting out of floating-raft-enriched fractions upon PKC-dependent stimulation (Fig. 6I,P). Moreover, Kv1.3(T523X) did not cluster in the presence of PSD-95 under basal conditions, and it underwent PKC-dependent internalization (Fig. 6J–O, circled cell).

### PKC-dependent Kv1.3 endocytosis targeted the channel for lysosomal degradation

**Figure 7:**
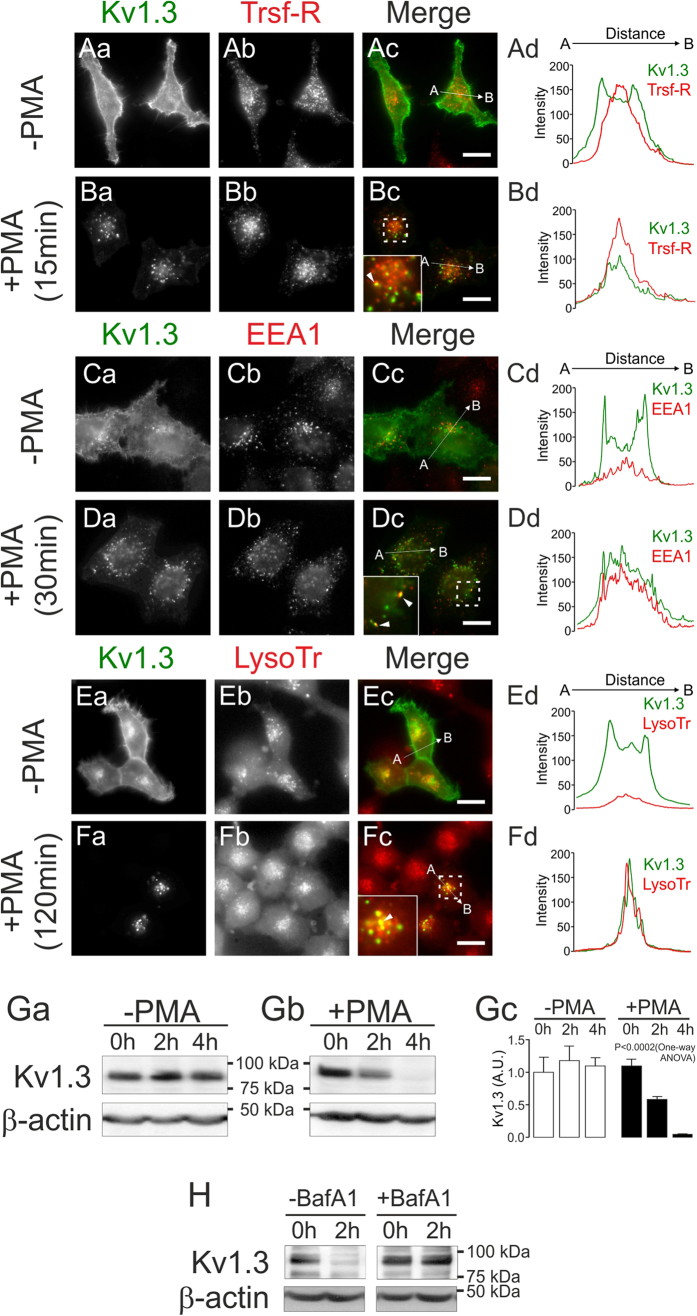
PKC-dependent endocytosis targets Kv1.3 to lysosomal degradation. HEK-293 cells stably expressing Kv1.3 were incubated with 1 μM PMA at 37 °C, and the endocytic pathway was evaluated. (Aa–Bd) Cells were untreated (Aa–Ad) or treated (Ba–Bd) with 1 μM PMA for 15 min, and the colocalization of Kv1.3-YFP and the transferrin receptor (Trsf-R), used as a marker of CCP, was analyzed. (Ca–Dd) Cells were cultured for 30 min without (Ca–Cd) or with (Da–Dd) PMA, and the distribution of Kv1.3-YFP and EEA1 was studied. (Ea–Fd) Cells were incubated for 120 min in the absence (Ea–Ed) or the presence (Fa–Fd) of PMA. The localization of Kv1.3-YFP into lysosomes was confirmed by LysoTracker (LysoTr) staining. Color code: Kv1.3, green; intracellular compartment marker, red. Yellow in merge panels (Ac, Bc, Cc, Dc, Ec, Fc) indicates colocalization. Insets in panels Bc, Dc and Fc magnify specific regions of interest, indicated by square outlines. Arrowheads indicate colocalizing structures. Bars represent 10 μm. (Ad, Bd, Cd, Dd, Ed, Fd) Histograms showing the results of the pixel-by-pixel analysis of the section A-B indicated by the arrow in the merged images. Color code: green, Kv1.3; red, marker (Trsf-R, EEA1 or LysoTr). Confocal images are representative from >25 cells analyzed in 3–5 independent experiments. (Ga–Gc) PKC-dependent PMA stimulation decreases Kv1.3 expression. Cells were pre-incubated for 3 h with 100 μg/ml of CHX in the absence (−, Ga) or the presence (+, Gb) of PMA, and the Kv1.3 protein expression was analyzed at the indicated times. (Gc) Quantification of Kv1.3 abundance, in arbitrary units (A.U.), from results represented in Ga and Gb. Values are mean ± SE of 4 independent experiments. Statistical analysis was performed by using One-Way ANOVA (P < 0.0002, +PMA vs time) with a Tuckey post-test. (p < 0.05, +PMA 0 h vs + PMA 2 h and + PMA 2 h vs + PMA 4 h; p < 0.001 + PMA 0 h vs + PMA 4 h). (**H**) Further inhibition of the lysosomal function by the addition of 20 μM BafA1 to the CHX pre-incubation counteracted the PMA-dependent degradation of Kv1.3. A representative result from 3 independent experiments is shown.

Our results in macrophages and CY15 dendritic cells suggested that the abundance of Kv1.3 was decreased under ADO-dependent PKC stimulation (Fig. 1). Therefore, to study in more detail whether Kv1.3 clathrin-mediated internalization ends in a lysosomal-associated degradative intracellular compartment, the PKC-dependent Kv1.3 endocytic mechanisms were further analyzed. After 15 minutes, Kv1.3 colocalized with Transferrin Receptor (Trsf-R), which is used as a CCP marker (Fig. 7Aa–Bd). After 30 minutes of PMA incubation, Kv1.3 was distributed within early endosomes, identified by Early Endosomal Antigen 1 (EEA1) (Fig. 7Ca–Dd). Finally, 120 minutes of PKC activation targeted Kv1.3 to lysosomes stained with LysoTracker^®^ Red (Fig. 7Ea–Fd). Concomitantly, 4 hours of PMA incubation in the presence of 100 μg/ml CHX triggered a massive decrease of Kv1.3 expression (Fig. 7Ga–Gc). Further, when also inhibiting lysosomal degradation using the vacuolar-type H^+^ -ATPase inhibitor BafA1 (20 μM), the rapid PKC-associated decrease in Kv1.3 abundance was halted (Fig. 7H). Overall, our results suggest that PKC activation, either by ADO or by PMA, triggered a down-regulation of cell-surface Kv1.3 via CCP that targets the channel for lysosomal degradation.

### PKC activation induces ubiquitination-dependent Kv1.3 endocytosis mediated by Nedd4-2 ubiquitin ligase activity

**Figure 8:**
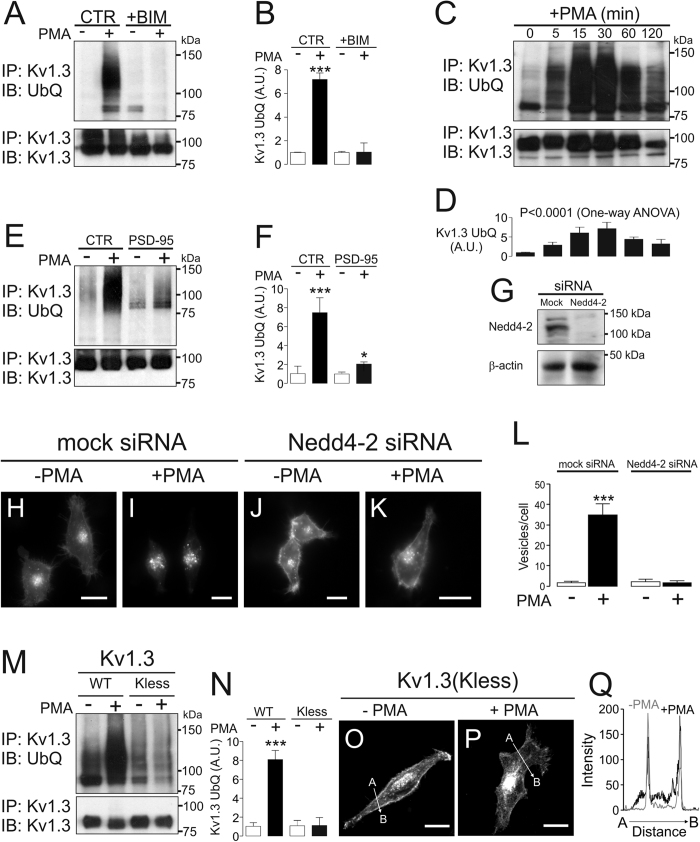
PKC-dependent internalization of Kv1.3 requires Nedd4-2-mediated ubiquitination of the channel. HEK cells with stable expression of Kv1.3-YFP were treated with 1 μM PMA for 30 min at 37 °C, and the ubiquitination of the channel was analyzed. (**A**,**B**) Ubiquitinated Kv1.3 expression in the absence (−) or the presence (+) of PMA with (+BIM) or without (CTR) BIM. (**C**,**D**) Time-course of Kv1.3 ubiquitination upon PMA incubation. Persistent PMA incubation was performed, and cell lysates were collected at the indicated times. (**E**,**F**) PSD-95 hampered the ubiquitination of Kv1.3 in the presence (+) of PMA. (**A**,**C**,**E**) Representative western blots. Total lysates immunoprecipitated against GFP (IP: Kv1.3) were immunoblotted using anti-GFP (IB: Kv1.3) or anti-ubiquitin (IB: UbQ) antibodies. (**B**,**D**,**F**) Expression of ubiquitinated Kv1.3 in arbitrary units (A.U.). Values are mean ± SE of 3–5 independent experiments. (**B**,**F**) White bars, −PMA; Black bars, +PMA. *, p < 0.05; ***, p < 0.001 vs −PMA, Student’s t test. (**D**) Values are mean ± SE of 3 independent experiments. Statistical analysis: p < 0.0001 vs time, One-way ANOVA. (**G**) HEK-293 cells were transfected either with siRNA scramble (Mock) or with siRNA against Nedd4-2 to deplete the Nedd4-2 protein expression. Total lysates were blotted with anti-Nedd4-2 and anti-β-actin antibodies. (**H**–**L**) The depletion of Nedd4-2 hampered the PKC-dependent PMA-induced Kv1.3 endocytosis. Confocal images are representative from >25 cells. (**L**) Quantification of Kv1.3 intracellular vesicles from representative images on panels H-K. Values are mean ± SE of >25 cells from 3 independent experiments. ***, p < 0.001 +PMA vs −PMA in mock transfected cells, Student’s t test. (**M**,**N**) The absence of lysines in the Kv1.3(Kless) mutant prevents the ubiquitination of the channel in the presence (+) and in the absence (−) of PMA. HEK-293 cells were transiently transfected with Kv1.3 WT (wild type) or Kv1.3(Kless), and the ubiquitination of the channel was analyzed in the absence (−) or the presence (+) of PMA (see materials and methods for details). (**M**) Total lysates immunoprecipitated for GFP (IP: Kv1.3) were immunoblotted with anti-GFP (IB: Kv1.3) or anti-ubiquitin (IB: UbQ). (**M**) Representative western blot from 4 independent experiments. (N) Expression of ubiquitinated Kv1.3 in arbitrary units (A.U.). Values are mean ± SE of 4 independent experiments. White bars, −PMA; Black bars, +PMA. ***, p < 0.001 vs −PMA in the Kv1.3 WT channel, Student’s t test. (**O**,**P**) Confocal images of HEK cells transfected with Kv1.3(Kless) in the presence (+, **O**) or the absence (−, **P**) of PMA. Kv1.3(Kless) was not internalized in the presence of PMA. Confocal images are representative from >25 cells. (**Q**) Histograms plotting the pixel-by-pixel analysis of the section A-B indicated by the arrow in panels O and P. Color code: grey, absence of PMA (−PMA); black, presence of PMA (+PMA). Note that in both cases Kv1.3 channels decorated the cell surface.

Ubiquitin-dependent endocytosis targets channels and transporters to lysosome-dependent degradation^22^,^39^,^40^. In this context, the E3 ubiquitin ligase Nedd4-2 associates with Kv1.3 and downregulates the activity of the channel^41^,^42^. Therefore, we next evaluated whether PKC-dependent Kv1.3 endocytosis, and thus lysosome-dependent degradation, was associated with the channel ubiquitination. PMA triggered a notable PKC-dependent ubiquitination of Kv1.3, which was hampered by BIM (Fig. 8A,B). Kv1.3 was transiently ubiquitinated upon PMA incubation and exhibited maximum levels at 15-30 minutes (Fig. 8C,D). Similar to Kv1.3 endocytosis and raft microdomain displacement, PSD-95 protected Kv1.3 against ubiquitination (Fig. 8E,F). To assess whether Nedd4-2 was involved in Kv1.3 endocytosis via PKC activation, we blocked its expression by using specific siRNA against Nedd4-2 (Fig. 8G). Kv1.3-YFP HEK cells transfected with Nedd4-2 siRNA were incubated with PMA for 30 min at 37 °C. When Nedd4-2 was depleted, the internalization of Kv1.3 was negligible (Fig. 8H–L). To definitively demonstrate the role of ubiquitination on the PKC-dependent Kv1.3 endocytosis, we mutated all the intracellular lysines of the channel (Lys70, 80, 146, 270, 342, 467, 476, 498, 519 and 520) to arginines. In the presence of PMA, the Kv1.3(Kless) mutant was not ubiquitinated (Fig. 8M,N). Relatedly, the internalization of Kv1.3(Kless) was minor upon PMA incubation (Fig. 8O–Q). Altogether, our results demonstrate that Kv1.3 is ubiquitinated by Nedd4-2 upon PKC-activation, triggering channel endocytosis and lysosome-dependent degradation.

## Discussion

Adenosine (ADO), which activates PKC signaling pathways, is an endogenous regulator in many physiological processes^7^,^8^,^43^,^44^. PKC modulates Kv1.3 activity, which is essential for a proper immune response^14^,^45^. Furthermore, both proteins are localized in the same membrane platforms, highlighting the importance of PKC-dependent regulation of Kv1.3 in leukocyte physiology^10^,^34^. Because Kv1.3 participates in the onset of several immunological diseases^3^,^46^, elucidating the mechanism by which ADO-dependent PKC-activation modulates this channel is essential for understanding the immune response. Therefore, we analyzed the PKC-based mechanisms that control the cell-surface levels of Kv1.3 as an efficient way to regulate the function of this ion channel. ADO, activating PKC, initiates a signaling cascade that modulates the cell-surface abundance of Kv1.3 by channel endocytosis. In addition, PSD-95 stabilizes Kv1.3 into lipid raft microdomains and protects the channel against internalization. Kv1.3 endocytosis is a CCP-dependent mechanism that drives the channel to lysosome-associated degradative intracellular compartments and requires previous ubiquitination by the E3 ubiquitin ligase Nedd4-2.

While activation of mononuclear phagocytes increases Kv1.3, anti-inflammatory agents decrease channel expression^14^,^16^,^47^. ADO, via A_2A_ and A_2B_ receptors, is a potent endogenous immunosuppressor^7^,^15^, and it decreases Kv1.3 levels in macrophages and dendritic cells. In addition, ADO induced internalization of the channel in HEK-293 cells, leading to PKC-mediated Kv1.3 lysosomal degradation. The presence of fewer channels at the cell surface results in less Kv1.3-driven signaling and thereby leads to immunosuppression in leukocytes^47^. In contrast, an ADO-mediated PKC-dependent mechanism stabilizes Kir6.2 channels at the myocyte plasma membrane^23^. However, PKC activation induces internalization of the epithelial sodium channel (ENaC) and the dopamine transporter (DAT) by the same clathrin-mediated endocytosis (CME) mechanism as Kv1.3^48^,^49^,^50^. CME also participates in the Kv1.2 and Kir1.1 endocytic pathways in neurons and renal cells^51^,^52^. Our results support that the down-regulation of Kv1.3 would trigger immunosuppression via PKC-dependent CME in leukocytes. This mechanism accelerates the degradation of the channel by targeting it to the lysosomal compartment after PKC-dependent stimulation. We further demonstrated that this process requires direct ubiquitination of Kv1.3 by the E3 ubiquitin ligase Nedd4-2. Recent evidence has shown that Nedd4-2 also drives Kv1.3 toward proteasomal degradation^41^. Thus, efficient Nedd4-2-dependent ubiquitination triggers the down-regulation of Kv1.3 via lysosomes and proteasomes, likely acting as redundant and complementary pathways. PKC activation triggers endocytosis and ubiquitination of proteins such as DAT, the glutamate transporter GLT1, the cationic amino acid transporter CAT-1 and aquaporin-2 (AQP2)^22^,^50^,^53^,^54^. Nedd4-2 downregulates Kv1.3 currents, but no direct ubiquitination of the channel had been demonstrated until now^41^,^42^. The absence of a canonical PY motif together with the irrelevance of a SH3 signature and several lysines within the C-terminal domain of Kv1.3 would suggest alternative mechanisms of association^41^.

In T-cells and macrophages, Kv1.3 is targeted to lipid raft membrane microdomains, where it is concentrated upon cellular activation^11^,^55^. Altered raft-associated Kv1.3 localization in the IS occurs at the onset of the disease lupus erythematosus^38^ and a minor raft targeting of Kv1.3 modifies the physiological response^34^,^47^. We demonstrated that PKC activation displaced Kv1.3 from lipid rafts, similar to what was described for the transporter NET^56^. However, unlike that of NET, the PKC-dependent endocytosis of Kv1.3 is independent of caveolae/lipid raft internalization. Several ion channels, such as TRPV5 and Kir6.1, use the caveolae-dependent internalization machinery^35^,^57^. TRPV5 endocytosis is inhibited by caveolin-1 knockdown^57^. However, similar to the cationic amino acid and dopamine transporters (CAT1 and DAT, respectively), Kv1.3 was internalized via CCP and independently of caveolae/rafts^22^,^50^. In this context, the localization of Kv1.3 in and out of rafts would have important physiological consequences. Therefore, recruitment and/or stabilization of the channel at the proper location by accessory proteins, such as caveolins and MAGUKs, would influence the immune response. PKC and Kv1.3 are distributed into lipid raft membrane microdomains within T-lymphocytes, and they are recruited to the IS during T-cell activation^11^. Moreover, PKC and Kv1.3 are part of a signalplex that also includes the tyrosine kinase p56lck, adaptor proteins such as Kvβ2 and hDlg (human homolog of the Drosophila discs large tumor suppressor protein), PSD-95 (postsynaptic density 95), ZIP-1 (Zrt/Irt-like protein) and ZIP-2, and the accessory protein CD4^58^. This cluster facilitates the phosphorylation of Kv1.3, thereby modulating its function. Our results show that PSD-95 association redistributes Kv1.3 into less-mobile membrane microdomains compatible with lipid rafts. In T-lymphocytes, PSD-95 interacts with Kv1.3, inducing clustering and recruiting Kv1.3 into the IS^37^. Similarly, PSD-95 recruits Kv1.4 into lipid rafts, inhibiting the internalization of Kv1.4^59^. A dynamic partitioning model would explain the behavior of raft proteins, suggesting movements in and out of raft domains in a steady-state equilibrium. This spatial distribution would permit proteins to transiently populate raft domains as well as to undergo diffusion outside of rafts. It is tempting to speculate that Kv1.3 would exit rafts being internalized via CCP during immunosuppression. In fact, Kv1.5, acting as an immunosuppresor, influences Kv1.3 by displacing heteromeric Kv1.3/Kv1.5 channels out of rafts in macrophages^34^,^47^,^60^. In this scenario, PKC would enhance the exit of Kv1.3 from raft areas and/or facilitate its distribution into clathrin-accessible regions to be endocytosed. In fact, PMA-induced PKC-activation in T-lymphocytes inhibits Kv1.3-dependent K^+^ currents^6^. Therefore, a functional Kv1.3-PSD-95 interaction is required not only for correct Kv1.3 redistribution in the vicinity of IS formation but also to prevent a massive internalization of the channel as a consequence of PKC-dependent immunosuppressive insults.

New treatments targeting inflammation-mediated organ dysfunction associated with autoimmune diseases are worth investigation. After persistent activation, mononuclear phagocytes play a pivotal role, supporting the characterization of ADO as an immunomodulatory agent. Adenoreceptor stimulation attenuates inflammation-mediated damage by down-regulating phagocytic activity and preventing excessive respiratory burst activation. For example, the inflammatory response is partially responsible for the damage associated with reperfusion of ischemic tissues. ADO modulation has been demonstrated in ischemia/reperfusion injury^61^. In this respect, selective inhibition of A_2B_ enhances intestinal inflammation and injury following ischemia/reperfusion, whereas specific A_2B_ agonist treatment protects against intestinal injury^62^. A_2B_ receptor agonists reduce myocardial ischemia/reperfusion damage by promoting anti-inflammatory M2 macrophages together with decreases in M1 macrophage and neutrophil infiltration in re-perfused hearts^63^. In this context, Kv1.3 is a viable pharmacological target for neuroinflammation associated with ischemia/reperfusion stroke^64^. Our results shed light on the molecular mechanism underlying the anti-inflammatory function of A_2B_ agonist therapy via the modulation of Kv1.3.

## Methods

### Expression plasmids and site-directed mutagenesis

The rat Kv1.3 in the pRcCMV construct was provided by T.C. Holmes (University of California, Irvine, CA). The channel was subcloned into pEYFP-C1 (Clontech). The rKv1.3 construct that was externally tagged with HA between S3 and S4 was obtained from D.B. Arnold (University of Southern California, CA). For some experiments, the HA-Kv1.3 channel was further subcloned into the pEYFP-C1 plasmid, generating the double-tagged HA-Kv1.3-YFP channel. All Kv1.3 mutants were generated in the pEYFP-Kv1.3 channel. Single and multiple Kv1.3 mutants were generated using the QuikChange site-directed and multi-site-directed mutagenesis kits (Stratagene). All mutations were verified using automated DNA sequencing. Myc-PSD-95 was a kind gift from Dr. F. Zafra (Centro de Biología Molecular Severo Ochoa, Madrid).

### Cell culture, transfections and incubations

Unless specified, all reagents were purchased from Sigma-Aldrich. HEK-293 and CY15 mouse dendritic cells were cultured in DMEM and RPMI culture medium (Lonza), respectively, supplemented with 10% fetal bovine serum (FBS), 10,000 U/ml penicillin, 100 μg/ml streptomycin and 2 mM L-glutamine (GIBCO). Murine bone marrow-derived macrophages (BMDM) were isolated and cultured as previously described^14^. HEK-293 cells were incubated with 200 μM Adenosine (ADO) or 1 μM phorbol-12-myristate-13-acetate (PMA) in DMEM for the specified times. BMDM and CY15 cells were incubated for 24 h with or without 200 μM ADO in the presence or the absence of 100 ng/ml of lipopolysaccharide (LPS). Experiments and surgical protocols were performed in accordance with the guidelines approved by the ethical committee of the Universitat de Barcelona and following the European Community Council Directive 86/609 EEC.

Transfection was performed using Metafectene^®^ Pro (Biontex) at approximately 80% confluence. Kv1.3-YFP experiments in HEK-293 cells were mainly performed using a cell line with stable Kv1.3-YFP expression. Briefly, 24 h after Kv1.3 transfection, cells were cultured in the presence of 500 μg/ml of G418 (Geneticin) for selection. Geneticin-resistant clones were maintained in the presence of 250 μg/ml G418. When required, transient transfections were performed 24 h to 48 h before the experiment. For the confocal analyses, cells cultured in the same medium were plated on poly-lysine-coated coverslips. Cells were incubated with 200 μM ADO or 1 μM PMA in DMEM for the specified times. DMSO (dilution 1:1,000) was used as a negative control. Cells were washed in PBS (without K^+^), fixed with 4% paraformaldehyde in PBS for 10 min and mounted with Aqua Poly/Mount from Polysciences, Inc. In some experiments, cells were pre-incubated for 15 min in the presence of 1 μM bisindolylmaleimide (BIM) as a PKC inhibitor or with DMSO as vehicle or for 3 h in the presence of 100 μg/ml cycloheximide (CXM) with or without 2 μM bafilomycin A1 (BafA1), as the inhibitor of the lysosomal function. When indicated, cells were pre-incubated with Filipin (5 μM), Nystatin (15 μM) and methyl-β-cyclodextrin (5 mM, MβCD) for 45 min. The caveolin-deficient HEK-293 cell line (Cav1^−^) was also used^12^.

### Protein extraction, co-immunoprecipitation, raft isolation and western blot analysis

Cells were washed twice in cold PBS before being lysed on ice with NHG solution (1% Triton X-100, 10% glycerol, 50 mmol/L HEPES pH 7.2, 150 mmol/L NaCl) supplemented with 1 μg/ml aprotinin, 1 μg/ml leupeptin, 1 μg/ml pepstatin and 1 mM phenylmethylsulfonyl fluoride to inhibit proteases. Total lysates were centrifuged at 16,000 × *g* for 15 min at 4 °C, and the protein content was measured using the Bio-Rad Protein Assay (Bio-Rad).

Samples were precleared with 30 μl of protein G-Sepharose beads for 2 h at 4 °C with gentle mixing as part of the co-immunoprecipitation procedures. Beads were removed by centrifugation at 1,000 × *g* for 30 s at 4 °C. Samples were incubated overnight with the specified antibody (4 ng/μg protein) at 4 °C with gentle agitation. Next, 30 μl of protein G-Sepharose 4 fast flow (GE Healthcare) was added to each sample, and the samples were incubated for 4 h at 4 °C. Beads were removed by centrifugation at 1,000 × *g* for 30 s at 4 °C, washed four times in NHG, and resuspended in 80 μl of SDS sample buffer.

Protein samples (50 μg) and immunoprecipitates were boiled in Laemmli SDS loading buffer and separated using 10% SDS-PAGE. Next, samples were transferred to nitrocellulose membranes (Immobilon-P, Millipore) and blocked in PBS supplemented with 5% dry milk and 0.05% Tween-20 before the immunoreaction. The filters were immunoblotted with antibodies against Kv1.3 (1/200, NeuroMab), iNOS (1/200, Santa Cruz), HA (1/200, Sigma), GFP (1/1,000, Roche), myc (1/1,000, Sigma), PAN-caveolin (1/250, BD Bioscience), clathrin heavy chain (1/500, BD Bioscience), dynamin II (1/1,000, ABR), total PKC (1/500, Santa Cruz), phosphorylated PKCε (1/200, Santa Cruz) and ubiquitin and Nedd4-2 (1/1,000, Santa Cruz) and β-actin (1/50,000, Sigma).

Lipid raft isolation was performed as previously described^34^. Briefly, samples were homogenized in MES (2-Morpholino ethanesulfonic acid)-buffered saline (24 mM MES, pH 6.5, and 0.15 mM NaCl) plus 1% Triton X-100 and centrifuged at 3,000 g for 5 min at 4 °C. Next, sucrose was added to achieve a final concentration of 40%. A 5–30% linear sucrose gradient was layered on top and further centrifuged (39,000 rpm) for 20–22 h at 4 °C in a Beckman SW41 Ti swinging bucket rotor. Gradient fractions (1 ml) were collected from the top and analyzed by Western blot.

### Confocal microscopy and subcellular compartment identification

Cells fixed with 4% paraformaldehyde in PBS for 10 min were further permeabilized using 0.1% Triton X-100 for 10 min. After a 60 min incubation with a blocking solution (PBS, 10% goat serum, 5% non-fat dry milk), cells were incubated for 60 min with anti-Clathrin (clathrin heavy chain, 1/100, BD Bioscience), anti-α subunit of AP2 (Adaptor-related Protein complex 2, 1/500, AP.6, American Type Culture Collection), anti-Transferrin receptor (1/1,000, Abcam) or anti-EEA1 (Early Endosomal Antigen 1, 1/1,000, BD Bioscience) in PBS, 10% goat serum and 0.05% Triton. For the extracellular distribution of HA-Kv1.3-YFP, cells were incubated with anti-HA (1/1,000, Sigma) under non-permeabilizing conditions. Next, cells were further incubated for 45 min with an Alexa Fluor secondary antibody (1:500, Molecular Probes) in PBS and BSA (2%). All experiments were performed at 21–23 °C (RT). In some experiments, cells were washed with PBS at 4 °C and stained with LysoTracker^®^ red (1/1,000, Molecular Probes) for 30 min at 4 °C. Staining with FITC-labeled cholera toxin β subunit (CTXβ) for lipid raft microdomains was performed under non-permeabilized conditions. Cells washed with PBS were stained with FITC-CTXβ for 30 min at 4 °C. Subsequently, cells were washed and fixed as above. Cells were examined with a 63x oil immersion objective on a Leica TCS SL laser-scanning confocal microscope. All offline image analyses were performed using a Leica confocal microscope, Image J software and Sigma Plot. The level of endocytosis was quantified analyzing the number of intracellular vesicle accumulation by using the automatic particle counting protocol of the Image J software and setting threshold around 75% to discard the membrane surface mask.

### siRNA transfections

Synthetic siRNAs for Clathrin, dynamin II and the missense negative control (Mock) were purchased from Dharmacon. Duplexes were resuspended in 1x siRNA universal buffer (Dharmacon) to 20 μM. HEK-293 cells expressing the stable Kv1.3-YFP channel were grown in six-well plates to 50% confluence. Cells were transfected with siRNA duplexes to a final concentration of 120 nM in 5 μl DharmaFECT1 reagent (Dharmacon, Inc). After 36 h, a second transfection was performed, and the cells were replated into 12-well plates on the following day for internalization experiments. The efficiency of knockdown was evaluated by Western blotting. Mock- and siRNA-transfected cells were processed for immunofluorescence as described above.

### Antibody-feeding endocytosis assay

Cells grown on glass coverslips were incubated with 1–2 μg/ml of anti-HA11 (1/1,000, Covance) in DMEM for 30–60 min at RT, washed twice and incubated at 37 °C in the presence or in the absence of 1 μM PMA for 30 min. The cells were then washed with ice-cold Ca^2+^/Mg^2+^ -free PBS (CMF-PBS) and fixed with freshly prepared 4% paraformaldehyde for 8 min at room temperature. Cells were stained with secondary anti-mouse antibody conjugated with Cy5 (5 μg/ml, saturating concentration) in CMF-PBS containing 0.5% BSA at RT for 60 min to occupy surface HA11. After washings, the cells were permeabilized by 10 min of incubation in CMF-PBS containing 0.1% Triton X-100 at RT and then incubated with the same secondary antibody conjugated with Cy3 (1 μg/ml, non-saturating concentration) for 60 min to stain internalized HA11. Both primary and secondary antibody solutions were precleared by centrifugation at 100,000 × g for 20 min. After staining, cells were washed, and the coverslips were mounted in Mowiol (Calbiochem).

### Electrophysiology

Whole-cell currents were recorded using the patch-clamp technique in the whole-cell configuration with a HEKA EPC10 USB amplifier (HEKA Elektronik). PatchMaster software (HEKA) was used for data acquisition. We applied a stimulation frequency of 50 kHz and a filter at 10 kHz. The capacitance and series resistance compensation were optimized. In most experiments, we obtained an 80% compensation of the effective access resistance. Micropipettes were made from borosilicate glass capillaries (Harvard Apparatus) using a P-97 puller (Sutter Instrument) and fire polished. The pipettes had a resistance of 2–4 MΩ. For the stably transfected HEK-293 cells, pipettes were filled with a solution containing the following (in mM): 120 KCl, 1 CaCl_2_, 2 MgCl_2_, 10 HEPES, 10 EGTA, 20 D-glucose (pH 7.3 and 280 mOsm/l). The extracellular solution contained the following (in mM): 120 NaCl, 5.4 KCl, 2 CaCl_2_, 1 MgCl_2_, 10 HEPES and 25 D-glucose (pH 7.4 and 310 mOsm/l). Cells were clamped at a holding potential of –60 mV. To evoke voltage-gated currents, cells were stimulated with 250 ms square pulses from –60 to +50 mV in 10 mV steps. To analyze the C-type inactivation of Kv1.3, a 5 s depolarizing pulse of +60 mV was applied. Electrodes for CY15 cells were filled with a solution containing the following (in mM): 84 K-aspartate, 36 KCl, 10 KH_2_PO_4_, 6 K_2_ATP, 5 HEPES, 5 EGTA, and 3 MgCl_2_, pH 7.2. The extracellular solution contained the following (in mM): 136 NaCl, 4 KCl, 1.8 CaCl_2_, 1 MgCl_2_, 10 HEPES, and 10 D-glucose, pH 7.4. CY15 cells were clamped to a holding potential of −60 mV. To evoke voltage-gated currents, cells were stimulated with 250 ms square pulses ranging from –60 to +80 mV in 10 mV steps. The peak amplitude (pA) was normalized using the capacitance values (pF). Data analysis was performed using FitMaster (HEKA) and Sigma Plot 10.0 software (Systat Software). All recordings were performed at RT.

### Statistics

Statistical analysis was performed where indicated by means of One-Way or Two-Way ANOVA with Tukey or Bonferroni post-test respectively, Mann-Whitney U test or Student *t* test by using GraphPad Prism 5 (Graphpad Software Inc.).

## Additional Information

**How to cite this article**: Martínez-Mármol, R. *et al*. Ubiquitination mediates Kv1.3 endocytosis as a mechanism for protein kinase C-dependent modulation. *Sci. Rep.* **7**, 42395; doi: 10.1038/srep42395 (2017).

**Publisher's note:** Springer Nature remains neutral with regard to jurisdictional claims in published maps and institutional affiliations.

## Supplementary Material

Supplementary Information
