# Supplementary material for: Ubiquitination mediates Kv1.3 endocytosis as a mechanism for protein kinase C-dependent modulation
Source: Sci Rep. 2017 Feb 10;7:42395. doi: 10.1038/srep42395 (PMC5301257; doi:10.1038/srep42395)
Supplement: Supplementary Information [file srep42395-s1.pdf]

Supplementary Information

Ubiquitination mediates Kv1.3 endocytosis as a mechanism for protein kinase C-dependent modulation

Ramón Martínez-Mármol, Katarzyna Styrczewska, Mireia Pérez-Verdaguer, Albert Vallejo-Gracia, Núria Comes, Alexander Sorkin and Antonio Felipe

Legend to Supplementary Figures

Supplementary Figure 1. PMA-dependent Kv1.3 endocytosis in HEK-293 cells. Confocal images of HEK cells transfected with HA-Kv1.3-YFP. Antibody-feeding endocytosis assay targeting the Kv1.3-HA external epitope. Live cells, incubated with anti-HA antibody for 1 h at 4°C, were further treated with (+PMA) or without (-PMA) PMA for 30 min at 37°C. HA-Kv1.3-YFP distribution in the absence (A-D) or the presence (E-H) of PMA. (A, E) Total HA-Kv1.3-YFP channel distribution. (B, F) Extracellular Cy5-stained HA-Kv1.3. (C, G) Intracellular Cy3-stained HA-Kv1.3. PMA-dependent (G) and independent (C) HA-Kv1.3-YFP endocytosis. (D, H) Merge panels in the absence or the presence of PMA, respectively. Confocal images are representative from >25 cells analyzed in 3 independent experiments. Bars represent 10 µm.

Supplementary Figure 2. Caveolae/lipid raft-independent Kv1.3 endocytosis upon PMA incubation. The internalization of Kv1.3 in HEK-293 cells stably transfected with the channel was induced by 30 min of incubation with 1 µM PMA at 37ºC. (A-H) Cells were pretreated for 45 min without (A, E) or with 5 µM Filipin (B, F), 15 µM Nystatin (C, G) or 5 mM MβCD (D, H) prior to incubation with PMA. (I-L) HEK-293 cells with lentiviral depletion of caveolin 1 ($Cav1^-$) were transfected with Kv1.3-YFP and preincubated with 5 mM MβCD for 45 min before treatment with (+) or without (-) 1 µM PMA at 37ºC. Confocal images are representative from >25 cells analyzed in 3 independent experiments. Bars represent 10 µm.

Supplementary Figure 3. PSD-95 protects Kv1.3 against PMA-dependent endocytosis. HEK-293 cells were cotransfected with HA-Kv1.3-YFP and PSD-95-myc and treated with 1 µM PMA for 30 min at 37ºC. (A-F) Cells were incubated without (-, A-C) or with (+, D-F) PMA. $Kv1.3_{TOTAL}$ (green) stands for the Kv1.3-YFP signal. $Kv1.3_{EXTRACEL}$ (red) refers to the extracellular HA staining under non-permeabilizing conditions. Colocalization (yellow) decorates the membrane in the merge panel without PMA (C) but not in (F). (G-N) PSD-95 preserved $Kv1.3_{EXTRACEL}$ in the presence of PMA. Color code in merge panels: green, $Kv1.3_{TOTAL}$; blue, $Kv1.3_{EXTRACEL}$; red, PSD-95; cyan, colocalization between $Kv1.3_{TOTAL}$ and $Kv1.3_{EXTRACEL}$; white, triple colocalization. (O-V) PSD-95 counteracted the targeting of Kv1.3 to early endosomes (EEA1) in the presence of PMA. Color code in merge panels: green, Kv1.3-YFP; red, EEA1; yellow, colocalization between Kv1.3 and EEA1. PSD-95 is not shown in the merge panels. Circled cells in S-U are not transfected with PSD-95-myc. Insets in panel V show distinct colocalization of Kv1.3 and EEA1 in the absence (cells in circle) or the presence of PSD-95-myc. Confocal images are representative from >25 cells analyzed in 3 independent experiments. Bars represent 10 µm.

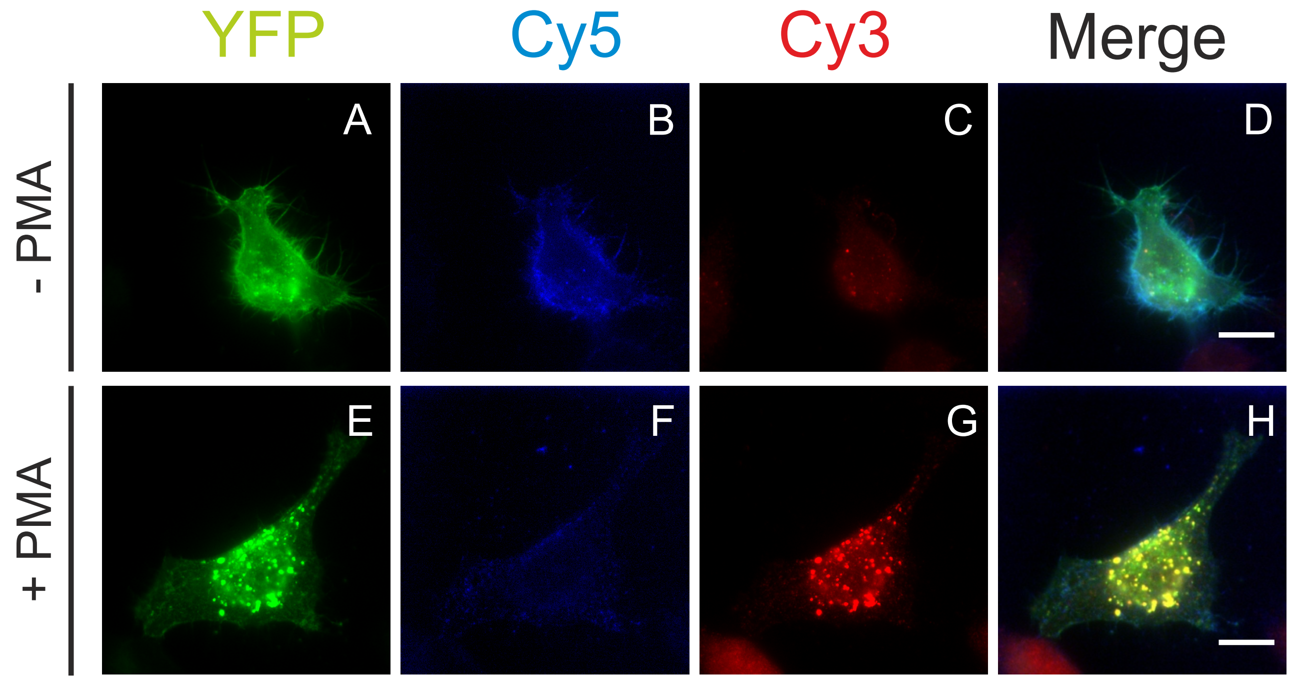

Figure S1

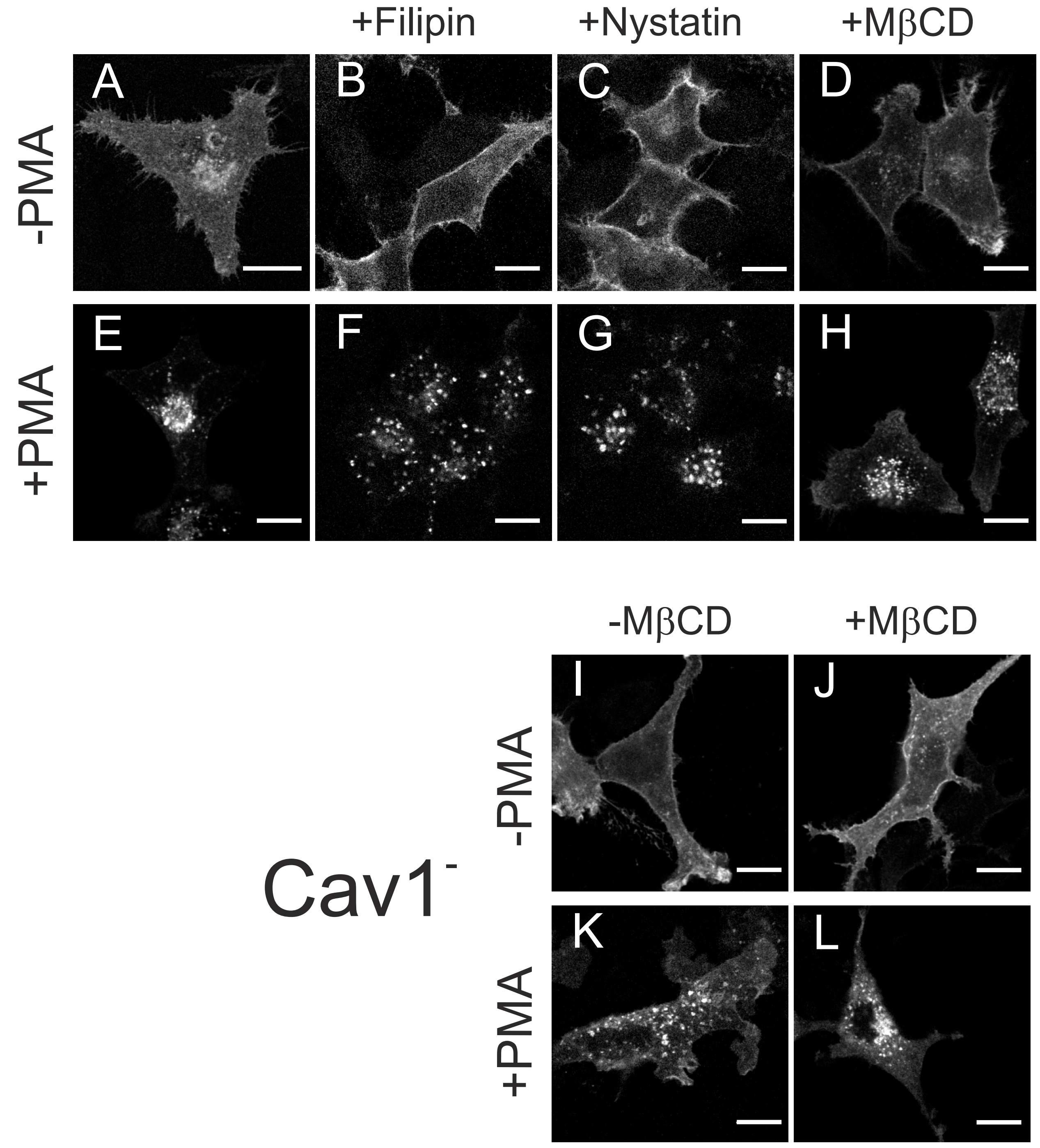
+Filipin
+Nystatin
+MβCD
-PMA
+PMA
A
B
C
D
E
F
G
H
-MβCD
+MβCD
Cav1-
-PMA
+PMA
I
J
K
L

Figure S2

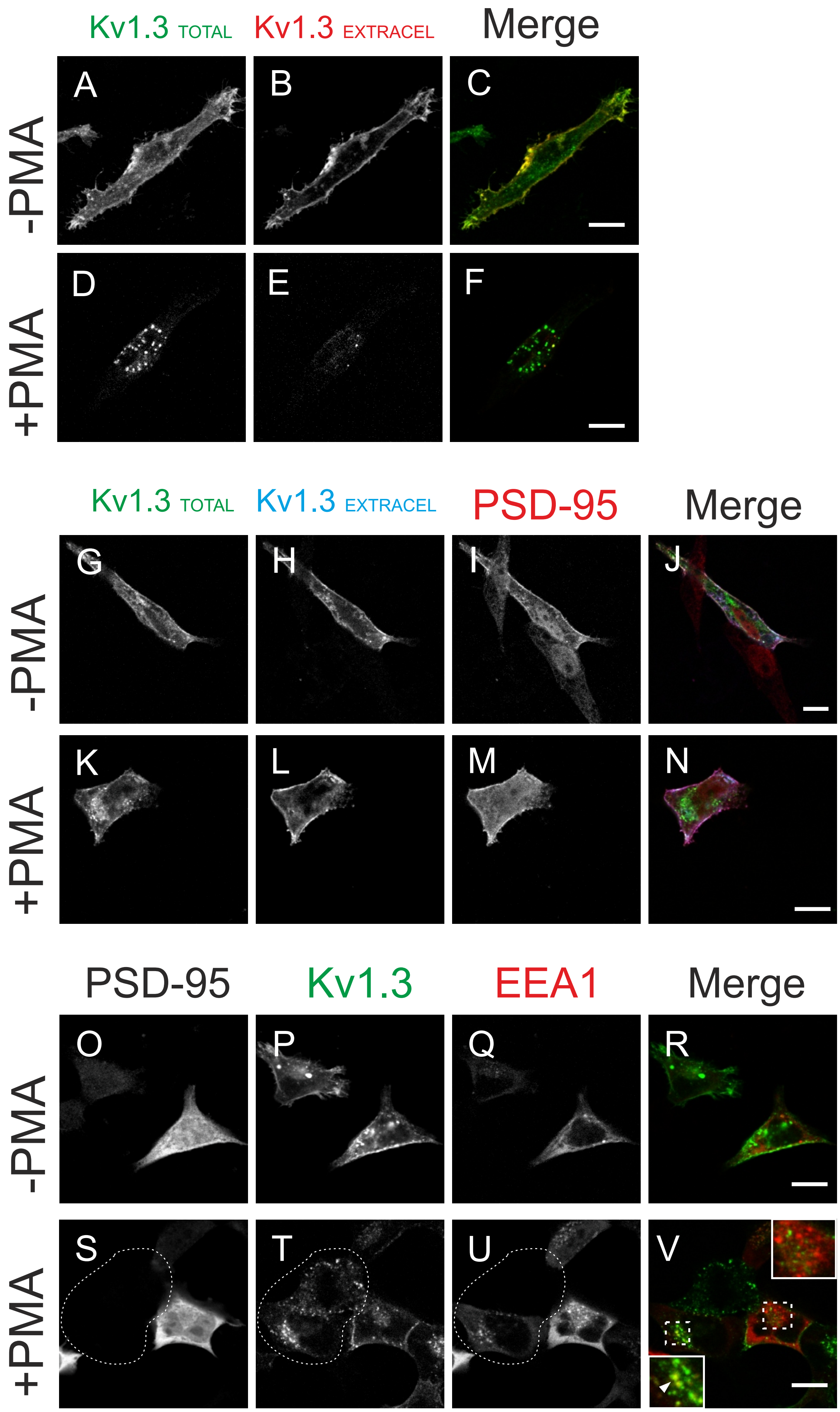
Kv1.3 TOTAL
Kv1.3 EXTRACEL
Merge
-PMA
+PMA
A
B
C
D
E
F
Kv1.3 TOTAL
Kv1.3 EXTRACEL
PSD-95
Merge
G
H
I
J
K
L
M
N
PSD-95
Kv1.3
EEA1
Merge
O
P
Q
R
S
T
U
V

Figure S3
